# Abscopal-like antitumor effect induced by localized low-temperature plasma application of normal tissue in mice

**DOI:** 10.1093/jrr/rraf077

**Published:** 2025-12-18

**Authors:** Ryo Ono, Ryota Sumitomo, Kengo Wada, Reima Jinno, Hideyuki Yanai

**Affiliations:** Department of Advanced Energy, The University of Tokyo, 5-1-5 Kashiwanoha, Kashiwa, Chiba 227-8568, Japan; Department of Advanced Energy, The University of Tokyo, 5-1-5 Kashiwanoha, Kashiwa, Chiba 227-8568, Japan; Department of Advanced Energy, The University of Tokyo, 5-1-5 Kashiwanoha, Kashiwa, Chiba 227-8568, Japan; Department of Advanced Energy, The University of Tokyo, 5-1-5 Kashiwanoha, Kashiwa, Chiba 227-8568, Japan; Department of Inflammology, Research Center for Advanced Science and Technology, The University of Tokyo, 4-6-1 Komaba, Meguro-ku, Tokyo 153-8505, Japan

**Keywords:** abscopal effect, normal tissue treatment, plasma treatment, immune response

## Abstract

We have previously reported that the localized application of gaseous plasma to normal tissues suppresses distant tumor growth in mice, resembling the abscopal effect of radiotherapy. Plasma, a partially ionized gas generated by a high-voltage electrical discharge, is fundamentally distinct from ionizing radiation and produces diverse reactive oxygen and nitrogen species that interact with biological tissues. This study examined the abscopal-like effects of normal tissue plasma treatment in BALB/c mice with subcutaneous Colon 26 tumors. The left dorsal skin, 2–3 cm from the tumor, was exposed to plasma for 10 min per day for 5 consecutive days, which delayed the growth of distant tumors. Similar tumor suppression was observed with abdominal exposure, indicating that the effect was not site-specific. In C.B-17 SCID mice (lacking T and B cells) and BALB/c nu/nu mice (lacking T cells), dorsal treatment did not suppress tumor growth, suggesting that T cells are likely involved in the response. Flow cytometric analysis of tumor-infiltrating immune cells in BALB/c mice revealed significant reductions in macrophages and increases in monocytes, with a possible but nonsignificant increase in dendritic cells. No consistent changes were detected in CD8^+^ T-cell proportion or ICOS (inducible T-cell costimulatory) expression. However, the lack of antitumor effects in immunodeficient mice suggests that CD8^+^ T cells are involved.

## INTRODUCTION

The abscopal effect is a rare phenomenon in radiotherapy, wherein local irradiation induces antitumor effects at distant, non-irradiated tumor sites [1]. This effect results from irradiation-induced immunogenic cell death (ICD) in cancer cells, leading to the release of damage-associated molecular patterns (DAMPs). DAMPs activate dendritic cells, which subsequently prime tumor-specific T cells that can attack tumors at remote sites [2, 3]. Recent reviews have suggested that the combination of radiotherapy with immune checkpoint inhibitors increases the likelihood of abscopal effects. Nevertheless, such events remain uncommon, likely due to factors such as tumor type, intrinsic tumor biology and host immune competence [4–8]. Despite these advances, the underlying mechanisms remain unclear.

We have previously demonstrated that gaseous plasma treatment of tumors in mice can induce an abscopal effect [9]. Plasma, generated by high-voltage electrical discharge, is fundamentally distinct from ionizing radiation. Notably, we also found that plasma exposure of normal tissues without direct tumor treatment can elicit such effects in tumor-bearing mice [10]. This finding is of particular interest in radiotherapy research, because comparable events are exceedingly rare in radiation treatment.

Plasma is a partially ionized gas composed of electrons, ions, reactive oxygen and nitrogen species (e.g. hydroxyl radicals, superoxide, and nitric oxide) and various excited species. It is generated through high-voltage electrical discharges in gasses, such as air or argon, where electrons accelerated by strong electric fields collide with atoms and molecules, producing charged particles and reactive species. These components can induce diverse biological responses, including cell cytotoxicity and immune activation [11, 12].

Plasma has been investigated for various medical applications, including cancer treatment, wound healing, sterilization, hemostasis, tissue regeneration and gene transfer [13–16]. In cancer treatment, numerous *in vitro* studies have shown that plasma exposure induces apoptosis in cancer cells [17–19], whereas *in vivo* mouse models have demonstrated tumor growth suppression following direct plasma application [20, 21]. Our group further reported that plasma treatment at the tumor resection site reduces local recurrence in mice [22].

Additionally, plasma has been shown to induce ICD and potentially activate systemic antitumor immunity [23–27]. Using a tumor rechallenge model, we demonstrated that plasma treatment generates durable systemic antitumor effects that are likely mediated by immune memory [28]. Most studies on immune activation have focused on direct plasma application to tumors, with only a few reports [10] describing the systemic effects following plasma treatment of normal tissues.

This study investigated the abscopal-like effects of plasma treatment on normal tissues. Using immunodeficient mice, we evaluated the role of the immune system in this response, and in wild-type mice, we used fluorescence-activated cell sorting (FACS) to profile plasma-induced changes in immune cell populations. We also assessed whether the site of plasma application influenced the induction of systemic antitumor effects.

Unlike ionizing radiation, the biological effects of plasma are largely confined to directly treated surfaces, because plasma does not inherently penetrate living tissues and functions as a surface treatment. For instance, in three-dimensional collagen matrices encapsulating A549 lung cancer cells, plasma treatment caused cell death only within a few millimeters of the treated site in radius and ~0.3 mm in depth [29].

## MATERIALS AND METHODS

### Plasma device

**Fig. 1:**
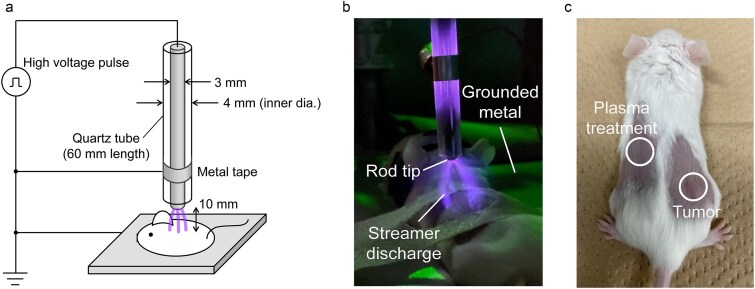
(a) Experimental setup. (b) Streamer discharge applied to the left dorsal skin of a mouse. (c) Locations of the subcutaneously implanted Colon 26 tumor and plasma treatment sites.

The plasma device comprised a glass tube (inner diameter: 4 mm) containing a concentrically positioned metal rod electrode (3 mm in diameter) with a hemispherical tip. High-voltage pulses (25 kV peak, 20 ns duration) were applied to the rod electrode at a repetition rate of 100 pulses/s (Fig. 1a). A grounded metal tape was wrapped around the quartz tube to generate an additional discharge inside the tube, thereby stabilizing the streamer discharge. As shown in Fig. 1b, pulsed repetitive discharges were generated between the hemispherical electrode tip and mouse skin, with the mouse placed on a grounded metal plate. The discharge comprised several thin filaments, which are characteristic of streamer discharge [30]. The discharge generates various reactive species [31] and induces apoptotic and necrotic cell death *in vitro* [22]. To maintain body temperature during treatment, a hot plate (37°C) was placed under the grounded plate.

Humidified oxygen gas was supplied through a glass tube at 0.5 L/min to enhance the generation of reactive oxygen species from the O_2_ and H_2_O molecules. Oxygen was humidified via a water bubbler, achieving >90% relative humidity at 23°C. The ambient air diffusing into the discharge region also contributes to the formation of reactive nitrogen species. The electrode tip-skin gap was maintained at 10 mm.

### Cells, mice and tumor inoculation

Colon 26 cells (RIKEN BioResource Center, Tsukuba, Japan) were cultured in RPMI medium supplemented with 10% fetal bovine serum and 1% penicillin–streptomycin at 37°C with 5% CO_2_. Female BALB/c, C.B-17 SCID (SCID) and BALB/c nu/nu mice (7-weeks-old) were obtained from Jackson Laboratory, Japan. SCID mice lack functional B and T lymphocytes, whereas BALB/c nu/nu mice are T-cell deficient. All mice were housed in an animal facility for 1 week before the experiments.

For tumor inoculation and plasma treatment, hair was removed from relevant skin areas using a depilatory cream. Mice were subcutaneously injected in the right limb with 2 × 10^5^ Colon 26 cells in 100 μl phosphate-buffered saline. A representative tumor appearance is shown in Fig. 1c. The mice were anesthetized with isoflurane during plasma treatment. All animal procedures were approved by and conducted in accordance with the guidelines of the Animal Ethics Committee of the University of Tokyo (A2023FS001 and RAC240003).

### Plasma treatment

For treatment, the left dorsal skin of the mice was exposed to plasma at a site 2–3 cm from the tumor (Fig. 1c). The experiments were conducted using BALB/c, SCID and BALB/c nu/nu mice. In a separate experiment using BALB/c mice, the plasma was applied to the mid-abdomen. Treatment was conducted for 10 min/day from days 6–10 (five consecutive days), with day 0 defined as the day of cancer cell inoculation. During treatment, the skin temperature increased by only 1°C [10], indicating negligible thermal effects. No visible inflammation or tissue damage was observed at treatment sites throughout the experiment. Tumor dimensions were measured using calipers, and volumes were calculated as (4/3)π × (length/2) × (width/2)^2^. Mice were randomly assigned to the control or plasma-treated groups prior to plasma exposure on day 6.

### FACS analysis

Tumor-infiltrating immune cell populations were analyzed using an LSRII Fortessa flow cytometer (BD Biosciences). The following fluorescent labeled antibodies were purchased from BioLegend or BD Biosciences: F4/80 (BM8), Ly6c (HK1.4), I-Ab (25–9-17), CD11b (M1/70), CD45 (30-F11), CD11c (N418), Ly6g (1A8), CD8 (53–6.7), B220 (RA3-6B2), NK1.1 (S17016D), CD4 (RM4–5) and ICOS (inducible T-cell costimulatory; C398.4A). Within the CD45^+^ fraction, the proportions of macrophages, monocytes, dendritic cells, CD8^+^ T cells, CD4^+^ T cells, NK cells and neutrophils were quantified, along with ICOS expression in CD8^+^ T cells. CD45^+^F4/80^+^ macrophages, CD45^+^F4/80^+^Ly6c^High^Ly6g^−^ monocytes, CD45^+^F4/80^+^Ly6c^−^Ly6g^+^ neutrophils, CD45^+^F4/80^+^CD11c^+^I-Ab^+^ dendritic cells, CD45^+^CD4^+^ CD4 T cells, CD45^+^CD8^+^ CD8 T cells, CD45^+^B220^+^ B cells and CD45^+^NK1.1^+^ NK cells were analyzed. Six independent experiments were performed, with measurements taken 0–4 days after completion of the 5-day plasma treatment (days 7–11 post tumor inoculation). In these analyses, the treatment schedule was shifted by 1 day compared with that of earlier experiments to ensure that tumor volumes at measurement were sufficient for accurate immune profiling. FlowJo software v10.7.2 (Tree Star) was used for data analysis.

## RESULTS

### Treatment of BALB/c mice

**Fig. 2:**
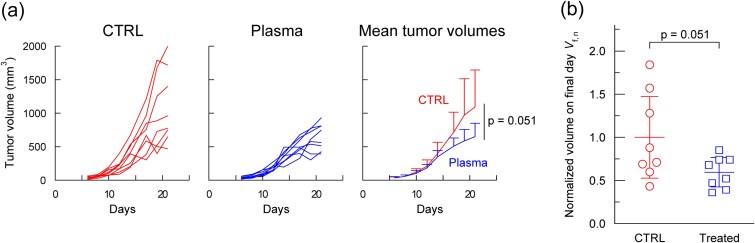
Left dorsal skin treatment in BALB/c mice. (a) Tumor growth curves for control and plasma-treated groups (*n* = 8). (b) Final-day tumor volumes (day 21) normalized to the mean tumor volume of the respective control group. Statistical analysis was performed using a *t*-test.

Plasma was first applied to the left dorsal skin of BALB/c mice (Fig. 1c) to establish baseline data for subsequent experiments (*n* = 8; Fig. 2a). Tumor volumes in the control and treated groups were comparable until approximately day 14, after which tumor growth in the treated group began to slow. This suggests that plasma exposure of normal tissue elicits an abscopal-like effect, although the difference was not significant (*t*-test, *P* = 0.051).

Our previous study indicated that a larger sample size may be necessary to achieve significance [10]. In that work, a significant effect (*P* < 0.01) was observed only in mice with tumor volumes at the start of treatment (*V*_s_, day 6) ≤ 70 mm^3^, with no significant effect in mice with *V*_s_ > 70 mm^3^. In the experiment shown in Fig. 2a, all mice satisfied this condition. Figure 2b shows the tumor volumes on the final day (day 21), normalized by the mean of the control group.

### Abdominal treatment of BALB/c mice

**Fig. 3:**
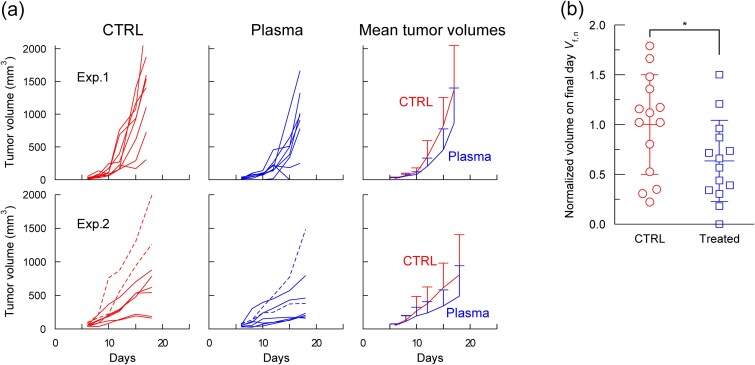
Mid-abdominal skin treatment in BALB/c mice. (a) Tumor growth curves for control and plasma-treated groups in two independent experiments, Exp. 1 and Exp. 2, (*n* = 8 each). Mice with tumor volumes at the start of plasma treatment (*V_s_*) > 70 mm^3^ are indicated by broken lines. (b) Final-day tumor volumes (day 17 for Exp. 1, day 18 for Exp. 2) normalized to the mean tumor volume of the control group in each experiment. Only mice with *V_s_* < 70 mm^3^ were included in this plot. Normalized data from both experiments were combined for statistical analysis using a *t*-test (^*^P < 0.05, *n* = 14).

To assess whether the abscopal-like effect was specific to dorsal treatment or could be induced from other normal tissue sites, the mid-abdomen of tumor-bearing BALB/c mice was treated with plasma. Two independent experiments (*n* = 8 per group) were performed to ensure reproducibility (Fig. 3a). Broken lines represent mice with *V*_s_ > 70 mm^3^ (i.e. not satisfying the criterion of *V*_s_ < 70 mm^3^). In both experiments, plasma treatment tended to slow tumor growth; however, the difference was not significant.

To improve statistical power, the results were combined after normalizing the tumor volumes. In Exp. 1, the tumor volume of each mouse on the final day (day 17) was divided by the mean tumor volume of the respective control group on the same day. The same procedure was applied in Exp. 2 using day 18 volumes. The normalized final day volumes (*V*_*f*, *n*_) from both experiments were combined. Only mice with *V_s_* < 70 mm^3^ were included in this analysis (*n* = 14; Fig. 3b). The mean of the control group was 1.0, whereas that of the treated group was 0.63, showing a significant reduction (^*^P < 0.05).

### Treatment of SCID mice

**Fig. 4:**
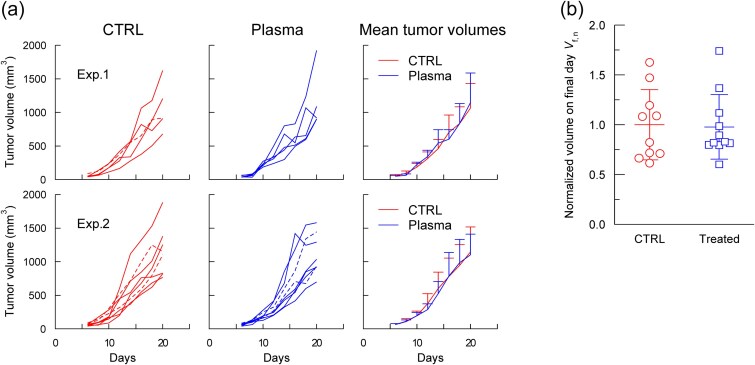
Left dorsal skin treatment in SCID mice. (a) Tumor growth curves for control and plasma-treated groups in two independent experiments: Exp. 1 (*n* = 5) and Exp. 2 (*n* = 8). Mice with *V_s_* > 70 mm^3^ are indicated by broken lines. (b) Final-day tumor volumes (day 20 for both experiments) normalized to the mean tumor volume of the control group in each experiment. Only mice with *V_s_* < 70 mm^3^ were included in this plot. Normalized data from both experiments were combined for statistical analysis using a *t*-test (*n* = 10 for control, *n* = 11 for treated).

To determine the role of adaptive immunity in the plasma-induced abscopal-like effect, experiments were performed using SCID mice lacking functional T and B lymphocytes. Plasma was applied to the left dorsal skin of the tumor-bearing mice in two independent experiments (*n* = 5 and *n* = 8 for Exps. 1 and 2, respectively). As shown in Fig. 4a, the tumor growth curves for the plasma-treated and control groups were nearly identical in both experiments. The normalized final day volumes (*V*_*f*, *n*_) are shown in Fig. 4b (*n* = 10 for control, *n* = 11 for treated). Both groups exhibited comparable means and variances, indicating no measurable antitumor effects in the SCID mice. These results suggest that adaptive immunity is essential for the systemic antitumor effects observed in immunocompetent BALB/c mice.

### Treatment of BALB/c nu/nu mice

**Fig. 5:**
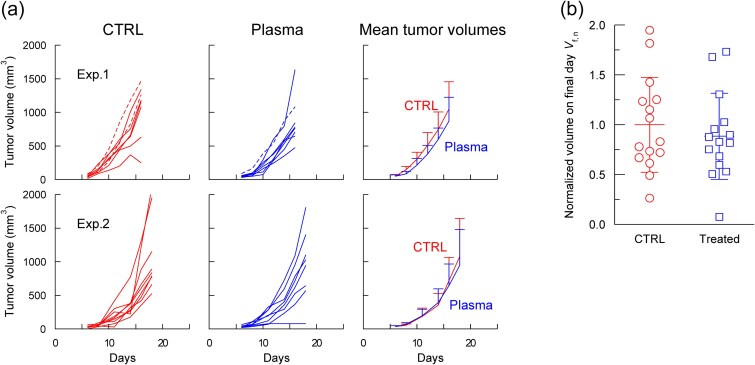
Left dorsal skin treatment in BALB/c nu/nu mice. (a) Tumor growth curves for control and plasma-treated groups in two independent experiments: Exp. 1 (*n* = 8) and Exp. 2 (*n* = 8 for treated, *n* = 9 for control). Mice with *V_s_* > 70 mm^3^ are indicated by broken lines. (b) Final-day tumor volumes (day 16 for Exp. 1, day 18 for Exp. 2) normalized to the mean tumor volume of the control group in each experiment. Only mice with *V_s_* < 70 mm^3^ were included in this plot. Normalized data from both experiments were combined for statistical analysis using a *t*-test (*n* = 15).

To further assess the role of T and B cells, experiments were conducted using BALB/c nu/nu mice, which lack functional T cells but retain B cell function. Plasma was applied to the left dorsal skin of the tumor-bearing mice in two independent experiments (*n* = 8 each, except *n* = 9 for the control group in Exp. 2). In Exp. 2, tumor growth was nearly identical between the control and treated groups (Fig. 5a). In Exp. 1, a modest growth delay was observed in the treated group; however, this difference was small. The normalized final day volumes (*V*_*f*, *n*_) obtained from both experiments are shown in Fig. 5b (*n* = 15). Although the mean tumor volume in the treated group was slightly smaller than in the control group, the difference was not significant, and the effect observed in Exp. 1 was not reproduced in Exp. 2. Considering that significant tumor suppression occurred in immunocompetent BALB/c mice but not in T cell–deficient BALB/c nu/nu mice, these findings suggest that the abscopal-like effect is largely dependent on T cells. Nevertheless, because nu/nu mice retain B cell function and also possess NK cells, the possible contribution of these cell types cannot be excluded.

### FACS analysis

**Fig. 6:**
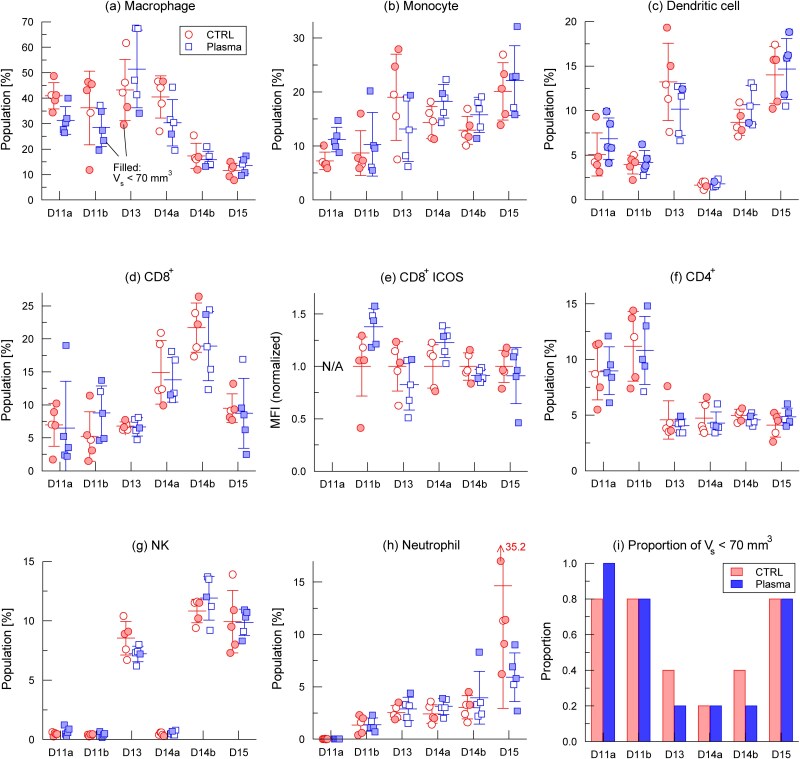
FACS analysis of tumor-infiltrating immune cell populations after plasma treatment in six independent experiments (D11a, D11b, D13, D14a, D14b, D15; *n* = 5 each). Data are shown for (a) macrophages, (b) monocytes, (c) dendritic cells, (d) CD8^+^ T cells, (e) ICOS expression on CD8^+^ T cells, (f) CD4^+^ T cells, (g) NK cells and (h) neutrophils within the CD45^+^ cell fraction. Mice with tumor volumes at the start of plasma treatment (*V_s_*) < 70 mm^3^ are indicated by filled symbols. (i) Proportion of mice with *V_s_* < 70 mm^3^.

**Figure 7:**
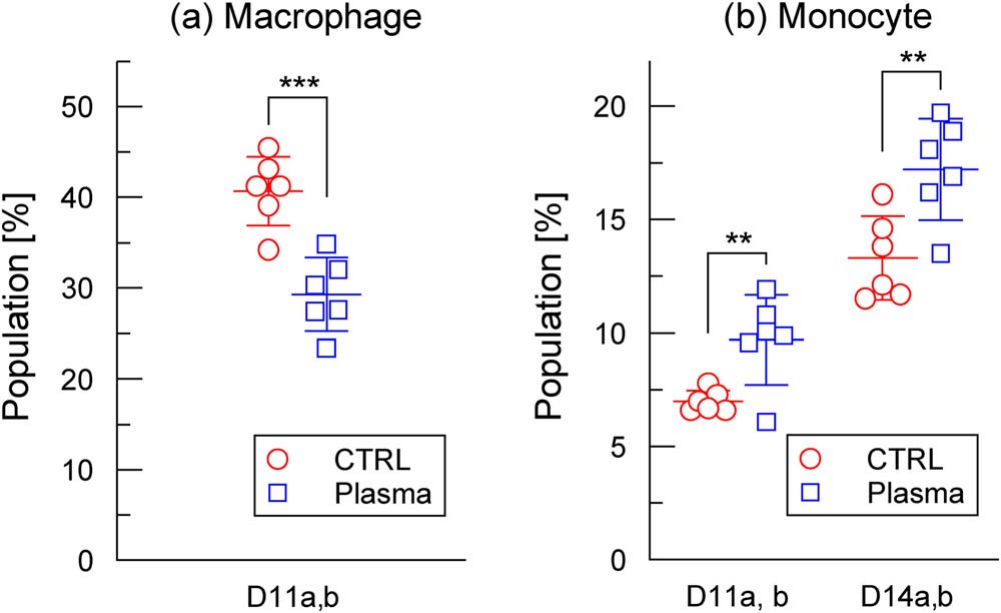
Statistical analysis of (a) macrophages and (b) monocytes from Fig. 6a and b. For macrophages, data from D11a and D11b were used. In each experiment, the highest and lowest values within each group were removed to reduce the influence of extreme outliers. Data from D11a and D11b were combined before analysis. For monocytes, the same outlier-removal procedure was applied. D11a/D11b and D14a/D14b were each combined for analysis (^***^P < 0.001, ^**^P < 0.01).

FACS analysis was performed in six independent experiments to profile tumor-infiltrating immune cell populations after plasma treatment. Each experiment was performed on a specific day after tumor inoculation: D11a and D11b (two separate experiments on day 11), D13 (day 13), D14a and D14b (two separate experiments on day 14), and D15 (day 15). All the experiments used five mice per group (*n* = 5). The five-day plasma treatment was scheduled such that measurements were obtained within 0–4 days of the final treatment. For example, D11a and D11b measurements were taken immediately or shortly after treatment completion, enabling the detection of early immune changes. The overall results are summarized in Fig. 6, and the proportion of mice that met the criterion of *V_s_* < 70 mm^3^ is specifically shown in Fig. 6i. This proportion could not be controlled experimentally and varied considerably among the experiments. In addition to mice with *V_s_* ≤ 70 mm^3^, those with *V_s_* > 70 mm^3^ were also included in the analysis to examine whether the tumor volume threshold affected plasma-induced immune modulation. In Fig. 6a–h, mice with *V*_s_ < 70 mm^3^ were indicated by filled symbols. In addition, statistical analyses were performed to evaluate the selective effects of plasma treatment on macrophages and monocytes, and the results are summarized in Table 1 and Fig. 7. A more detailed interpretation of these findings is provided in Discussion section.

**Table 1:** Proportion of mice with (a) lower macrophage or (b) higher monocyte populations, summarized across multiple experiments. For each experiment, the number of mice in the control (CTRL) and plasma-treated groups (*n* = 5 per group) was counted whose population (*P*) was lower (a) or higher (b) than the average value of the control group within the same experiment (*P*_CTRL,ave_). Counts were compiled separately for subgroups with initial tumor volume *V_s_* > 70 mm^3^ and *V_s_* ≤ 70 mm^3^. The macrophage analysis (a) includes data from D11a, D11b, D13 and D14a, while the monocyte analysis (b) includes D11a, D11b, D13, D14a, D14b and D15

(a) Macrophage (D11a ~ D14a)		
Number of mice with *P* < *P*_CTRL,ave_		
	CTRL	Plasma
*V_s_* > 70 mm^3^	4/9 (44%)	4/9 (44%)
*V_s_* ≤ 70 mm^3^	5/11 (45%)	11/11 (100%)
(b) Monocyte (D11a ~ D15)		
Number of mice with *P* > *P*_CTRL,ave_		
	CTRL	Plasma
*V_s_* > 70 mm^3^	6/13 (46%)	6/14 (43%)
*V_s_* ≤ 70 mm^3^	7/17 (41%)	13/16 (81%)

## DISCUSSION

FACS analysis showed that macrophages constituted a substantial fraction (30–50%) of CD45^+^ tumor-infiltrating cells through day 14, but dropped sharply to approximately 10% by day 15 (Fig. 6a). The marked difference between D14a and D14b suggests that this decline likely occurred rapidly around day 14. Tumor-associated macrophages (TAMs) are known to promote tumor progression [32]. Thus, during D11a–D14a, when macrophage levels remained high, TAMs may have exerted a strong pro-tumor influence.

Although this possibility is suggested, it should be noted that in this study macrophages were defined only by F4/80 expression and were not further subdivided into M1- and M2-polarized subtypes. Therefore, whether the observed reduction after plasma treatment corresponds specifically to TAMs (M2-polarized macrophages) remains uncertain. Further studies using additional markers such as CD206 will be required to clarify this issue.

In the D11a–D14a experiments, where macrophage levels were high, plasma treatment reduced these levels in most cases, except on D13. In the treated group, all *V_s_* < 70 mm^3^ mice (filled symbols) had macrophage levels below the control mean, whereas this pattern was absent in the controls. This suggests that plasma treatment consistently reduces macrophage abundance in small tumors. The treatment–control difference decreased by D14b and disappeared by D15. This suggests that the difference observed earlier diminishes with time, although it is also possible that the sharp decline in overall macrophage levels masks the treatment effect.

Table 1a quantifies this relationship, excluding D14b and D15 for the aforementioned reasons. Including these datasets complicates the assessment of plasma effects. For *V_s_* > 70 mm^3^ mice, the proportion of macrophages below the control mean was identical between the treated and control groups (44%), indicating no treatment effect. For *V_s_* < 70 mm^3^ mice, the control group proportion was 45%, similar to *V_s_* > 70 mm^3^, implying that tumor size alone did not alter macrophage abundance. In contrast, 100% of the mice treated with *V_s_* < 70 mm^3^ showed reduced macrophage levels, strongly indicating selective depletion by plasma treatment in this subgroup. In this study, mice with larger tumors (*V_s_* > 70 mm^3^) were included to verify whether the previously reported threshold [10] also applied to immune cell modulation, and the results confirmed that the changes were indeed more prominent in smaller tumors (*V_s_* ≤ 70 mm^3^).

To test statistical significance, we analyzed datasets from day 11 (D11a, D11b), which had high proportions of *V_s_* < 70 mm^3^ mice (Fig. 6i) and were therefore well suited for this purpose. D15, despite having a similar proportion, was excluded to avoid bias due to diminished differences. The highest and lowest values in each group were removed to limit the effects of extreme outliers, and the D11a/D11b data were combined (Fig. 7a). This analysis revealed a significant reduction in macrophage levels in the treated mice (^***^P < 0.001), consistent with suppression of tumor growth observed in Figs 2 and 3.

Monocyte levels (Fig. 6b) increased after plasma treatment in all experiments except on D13. Applying the same analysis as for macrophages (Table 1b), the proportion of mice exceeding the control group mean was similar between *V_s_* > 70 mm^3^ (46%) and *V_s_* < 70 mm^3^ (41%) controls, indicating no size-dependent baseline difference. In contrast, 81% of treated mice with *V_s_* < 70 mm^3^ exceeded the mean of the control group, suggesting that elevated monocyte levels may contribute to tumor suppression in this subgroup. Although the precise role of monocytes in tumor growth remains unclear, several studies have suggested that they can influence tumor progression [33].

The plasma-induced monocyte increase was statistically significant in the datasets from day 11 (D11a, D11b) and day 14 (D14a, D14b). Outlier-trimmed data from the same day were combined, and both day 11 and 14 datasets showed significant differences between the control and treated groups (Fig. 7b; ^**^P < 0.01).

Dendritic cell levels (Fig. 6c) increased after plasma treatment in all experiments, except on D13, but the differences from the controls were not statistically significant, even when restricted to mice with *V_s_* < 70 mm^3^. Given their role in initiating T-cell activation through antigen presentation [34], further studies with larger sample sizes and activation markers such as MHC class II, CD80, CD86 and CD40 will be important to clarify their contribution to plasma-induced tumor suppression.

Experiments in immunodeficient mice indicated that T cells likely contributed to the suppression of plasma-induced tumor growth (Figs 3 and 4). In contrast, FACS analysis did not reveal the expected changes in CD8^+^ T cells: their proportions increased through day 14 before declining on day 15, but no consistent differences were observed between control and treated groups, nor in ICOS expression (Fig. 6d and e). Thus, while the absence of antitumor effects in SCID and BALB/c nu/nu mice suggests a T-cell–dependent mechanism, this requirement was not clearly reflected in measurable changes in CD8^+^ T-cell abundance by FACS, even though T cells are well known to play a central role in systemic antitumor immunity [35]. Additional FACS studies incorporating activation markers and cytokine assays are needed to clarify the potential effects of plasma on CD8^+^ T cell function.

Other immune cell types, including CD4^+^ T cells, NK cells and neutrophils, showed no reproducible changes and were not analyzed further.

The D13 experiment displayed immune cell patterns that deviated from the other datasets, including increased macrophages, decreased monocytes and reduced dendritic cells after treatment, in contrast to the general trends. It also showed unusually high variability for these cell types and unusually low variability for CD8^+^ T cells, suggesting that uncontrolled experimental factors may have affected data reliability on D13.

The mechanisms by which plasma treatment of normal tissues modulates macrophage, monocyte and possibly dendritic cell populations remains unclear. The abscopal-like effect was not limited to dorsal skin exposure, as abdominal treatment in BALB/c mice yielded similar trends. *In vitro* work using three-dimensional collagen matrices with A549 lung cancer cells demonstrated that plasma-induced cytotoxicity is limited to a few millimeters laterally and ~0.3 mm in depth [29]. In the present *in vivo* study, plasma was applied to the skin, implying that both plasma-skin and subsequent skin-subcutaneous tissue interactions may be critical [36]. Notably, in previously reported ‘tumor-treated’ abscopal effects [9, 25], plasma was applied to the skin overlying subcutaneous tumors rather than directly to the tumor mass. The depth to which plasma-generated reactive species penetrate through skin remains uncertain, raising the possibility that such ‘tumor treatments’ mechanistically resemble normal-tissue treatment.

Potential contributions from remote physical factors generated by the plasma, such as electric fields, currents and electromagnetic radiation, cannot be excluded. These fields may act systemically during treatment, extending beyond the directly exposed sites. For example, *in vitro* studies have shown that plasma can inactivate cancer cells shielded by a 1 mm-thick polymer barrier [37], presumably via electric or electromagnetic penetration. Such non-contact propagation effects should also be considered when interpreting *in vivo* results of normal-tissue plasma treatment.

## CONCLUSION

This study examined the mechanisms underlying the abscopal-like effect induced by the plasma treatment of normal tissues, using immunodeficient mouse models and flow cytometric analysis of tumor-infiltrating immune cells. Plasma exposure reduced macrophage populations while increasing monocyte and potentially dendritic cell populations. These changes occurred preferably in mice with *V_s_* < 70 mm^3^, which is consistent with previous findings [10]. The absence of tumor growth suppression in SCID and BALB/c nu/nu mice suggests an important role for T cells, although no consistent changes were detected in CD8^+^ T-cell abundance or ICOS expression, and the precise mechanism remains unclear. An abscopal-like effect was observed following plasma exposure to either the dorsal or abdominal skin, indicating that it was not site-specific.

## Presentation at a conference

Parts of this research were presented at Spring Conferences of the Institute of Electrostatics Japan (IEJ) (2022, 2023, 2025), Annual Meeting of IEJ (2024), 2022 Gaseous Electronics Conference (2022), 35th International Conference on Phenomena in Ionized Gases (2023), and 82nd Annual Meeting of the Japanese Cancer Association (2023).
